# Professional

**Published:** 1870-01

**Authors:** 


### PROFESSIONAL.

At the recent meeting of the State Board of Dental Examiners, held at Columbus, the following persons appeared before the Board, passed a satisfactory examination, and each received a certificate of qualification to practice, viz: E. J. Way, Sandusky; W. P. Hall, Piqua; H. M. Reid, Cedarville; J. McKinley, Uhricksville; A. R. Lord, Shelby; S. J. Way, Waynesville.

These gentlemen are well known practitioners. None of them have been in practice less than twelve years, and some of them twenty-five. The fact that the older members of the profession are conforming to the requirements of the law, is very gratifying. It manifests an interest and confidence that ought to be entertained by every true member of the profession. Every one who has placed himself in a proper legal position can with propriety urge others to the same course; and he has a double interest in the faithful execution of the provisions of the law. Only in the fulfillment of the law will the good results sought by its enactment be realized by the public and the profession, while if it is permitted to remain a dead letter, the law making power and the profession are brought into contempt. T.

The annual meeting of the Ohio Dental College Association will be held in the College, Tuesday, March 1st, at 10 o'clock, A. M. As matters of importance will come before the meeting, it is desirable that every member be present. We hope all feel interested enough in the institution to take part in its management. T.

